# A Semi-Supervised Stacked Autoencoder Using the Pseudo Label for Classification Tasks

**DOI:** 10.3390/e25091274

**Published:** 2023-08-30

**Authors:** Jie Lai, Xiaodan Wang, Qian Xiang, Wen Quan, Yafei Song

**Affiliations:** 1 College of Air and Missile Defense, Air Force Engineering University, Xi’an 710051, China; 2 College of Air Traffic Control and Navigation, Air Force Engineering University, Xi’an 710051, China

**Keywords:** deep learning, semi-supervised learning, stacked autoencoder, pseudo label, regularization

## Abstract

The efficiency and cognitive limitations of manual sample labeling result in a large number of unlabeled training samples in practical applications. Making full use of both labeled and unlabeled samples is the key to solving the semi-supervised problem. However, as a supervised algorithm, the stacked autoencoder (SAE) only considers labeled samples and is difficult to apply to semi-supervised problems. Thus, by introducing the pseudo-labeling method into the SAE, a novel pseudo label-based semi-supervised stacked autoencoder (PL-SSAE) is proposed to address the semi-supervised classification tasks. The PL-SSAE first utilizes the unsupervised pre-training on all samples by the autoencoder (AE) to initialize the network parameters. Then, by the iterative fine-tuning of the network parameters based on the labeled samples, the unlabeled samples are identified, and their pseudo labels are generated. Finally, the pseudo-labeled samples are used to construct the regularization term and fine-tune the network parameters to complete the training of the PL-SSAE. Different from the traditional SAE, the PL-SSAE requires all samples in pre-training and the unlabeled samples with pseudo labels in fine-tuning to fully exploit the feature and category information of the unlabeled samples. Empirical evaluations on various benchmark datasets show that the semi-supervised performance of the PL-SSAE is more competitive than that of the SAE, sparse stacked autoencoder (SSAE), semi-supervised stacked autoencoder (Semi-SAE) and semi-supervised stacked autoencoder (Semi-SSAE).

## 1. Introduction

Deep learning has been a focus of machine learning research since it was proposed by Hinton et al. [1]. As a typical deep learning algorithm, the stacked autoencoder (SAE) [2] extracts hierarchical abstract features from samples by the autoencoder (AE), and then maps the abstract feature to the output by a classifier or regression algorithm. Compared with traditional neural networks, the multilayer structure of SAE represents a strong feature extraction capability, avoiding the limitations of traditional machine learning algorithms in manual feature selection [3]. Meanwhile, the greedy layer-wise training of the SAE determines the network parameters layer by layer and accelerates the convergence speed [4]. By virtue of excellent performance, the SAE has been applied to mechanical fault diagnosis [5,6], disease association prediction [7,8] and network intrusion detection [9,10].

The SAE has been extensively studied, and many methods of improvement have been introduced into the SAE. Vincent et al. [11] combined the SAE with the local denoising criterion and proposed the stacked denoising autoencoder (SDAE). Different from the SAE, the SDAE employs noise-corrupted samples to reconstruct noise-free samples, and it enhances the robustness of the abstract feature. To obtain a sparse feature representation, Ng et al. [12] integrated the sparsity constraint into the SAE and proposed the stacked sparse autoencoder (SSAE). The SSAE can reduce the activation of hidden nodes and use a few network nodes to extract representative abstract features. Masci et al. [13] proposed the stacked convolutional autoencoder (SCAE) by replacing the fully connected layer with convolutional and pooling layers to preserve the spatial information of the training images. By introducing the attention mechanism into the SAE, Tang et al. [14] constructed the stacked attention autoencoder (SAAE) to improve the feature extraction capability. Tawfik et al. [15] utilized the SAE to extract unsupervised features and merge the multimodal medical image. In addition, many other methods [16,17,18,19] have been proposed for the development and application of the SAE.

However, the manual labeling of large numbers of samples is impossible due to the limited knowledge and efficiency. In many fields, such as speech emotion recognition [20], medical image classification [21] and remote sensing image detection [22], the unprocessed training samples are usually only partially labeled, while the majority of samples are unlabeled. The supervised learning of the SAE requires sample labels to train the network and is unable to exploit the feature and category information contained in unlabeled samples, making it difficult to improve its generalization performance for the semi-supervised classification tasks. To tackle this problem, some studies in recent years have combined the SAE with semi-supervised learning. For the classification of partially labeled network traffic samples, Aouedi et al. [23] proposed the semi-supervised stacked autoencoder (Semi-SAE) to realize a semi-supervised learning of the SAE. This method needs unsupervised feature extraction for all samples in the pre-training stage and fine-tuning of the network parameters based on the classification loss of the labeled samples. By introducing the sparsity criterion into the Semi-SAE, Xiao et al. [24] proposed the seme-supervised stacked sparse autoencoder (Semi-SSAE). The Kullback–Leibler (KL) divergence regularization term added to the loss function improves the sparsity of the network parameters, and the Semi-SSAE is applied to cancer prediction. These improved SAE algorithms use only part of the information from the unlabeled samples in the feature extraction stage and have a limited generalization performance for semi-supervised classification tasks.

The pseudo label [25] is a simple and efficient method for implementing semi-supervised learning. It utilizes labeled samples to predict the class of unlabeled samples and integrates labeled and pseudo-labeled samples to train the network. Semi-supervised learning methods based on the pseudo label have been gradually applied to automatic speech recognition [26] and image semantic segmentation [27]. To overcome the limitations of the traditional supervised SAE and to improve the generalization performance, the pseudo label-based semi-supervised stacked autoencoder (PL-SSAE) is proposed by combining the SAE with the pseudo label. The PL-SSAE first stacks the AE to extract the feature information in all samples through layer-wise pre-training. Then, the supervised classification and iterative fine-tuning on the labeled samples are used for the class prediction of the unlabeled samples. Finally, the pseudo-label regularization term is constructed, and the labeled and pseudo-labeled samples are integrated to complete the training of the network. Different from the SAE and Semi-SAE, the PL-SSAE is able to exploit both feature information from unlabeled samples for feature extraction and category information for classification and fine-tuning, aiming to improve its semi-supervised learning performance. To the best of our knowledge, the PL-SSAE is the first attempt to introduce the pseudo label into the SAE, and it extends the implementation methods of the semi-supervised SAE.

The research contributions of this study can be summarized as follows:

- A new semi-supervised SAE named the PL-SSAE is proposed. By integrating the pseudo label with the SAE, the pseudo labels of the unlabeled samples are generated and the category information in the unlabeled samples is effectively exploited to improve the generalization performance of the PL-SSAE. The experimental results on various benchmark datasets show that the semi-supervised classification performance of the PL-SSAE outperforms the SAE, SSAE, Semi-SAE and Semi-SSAE.
- The pseudo-label regularization term is constructed. The pseudo-label regularization term represents the classification loss of the pseudo-labeled samples, and it is added to the loss function to control the loss balance between the labeled and pseudo-labeled samples and to prevent over-fitting.

The rest of this study is organized as follows. In Section 2, a brief introduction to the AE and SAE is described. In Section 3, the network structure and training process of the proposed PL-SSAE are detailed. In Section 4, the evaluation implementation and results on benchmark datasets are presented. In Section 5, the conclusion of this study is summarized.

## 2. Related Works

### 2.1. Autoencoder

**Figure 1:**
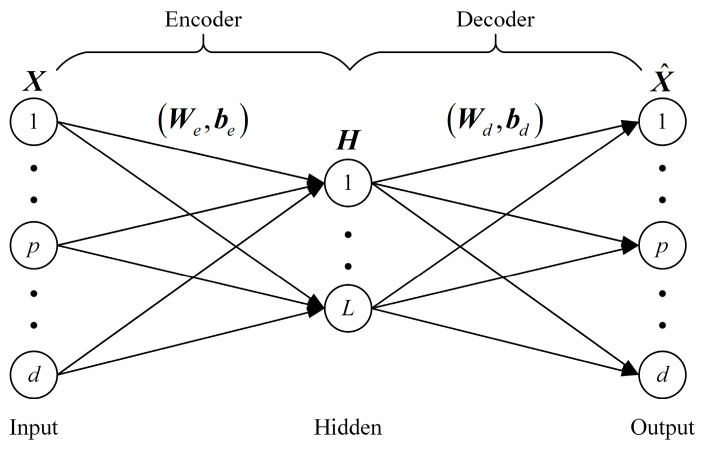
Network structure of the AE.

The AE is an unsupervised algorithm and consists of an encoder and a decoder. The encoder maps the input to the abstract representation and the decoder maps the abstract representation to the output. The network structure of the AE is shown in Figure 1.

For the input samples $X={\left\{x_{i}\right\}}_{i=1}^{N}$, the AE encodes the samples using linear mapping and a non-linear activation function:

(1) $$H=g\left(W_{e}X+b_{e}\right)$$

where $W_{e}$ is the weight matrix between the input layer and the hidden layer, $b_{e}$ is the bias of the hidden layer and g(⋅) is the activation function. The decoder completes the decoding of the abstract feature to receive the reconstructed samples:

(2) $$\hat{X}=g\left(W_{d}X+b_{d}\right)$$

where $W_{d}$ is the weights matrix between the hidden layer and the output layer and $b_{d}$ is the bias of the output layer. The AE requires an optimization algorithm to fine-tune the network parameters. The reconstruction error is minimized to learn representative abstract features in the samples. The loss function of the AE is formulated as follows:

(3) $$J_{AE}=\frac{1}{2}\sum_{i=1}^{n}{\left\|{\hat{x}}_{i}-x_{i}\right\|}_{2}^{2}$$

### 2.2. Stacked Autoencoder

**Figure 2:**
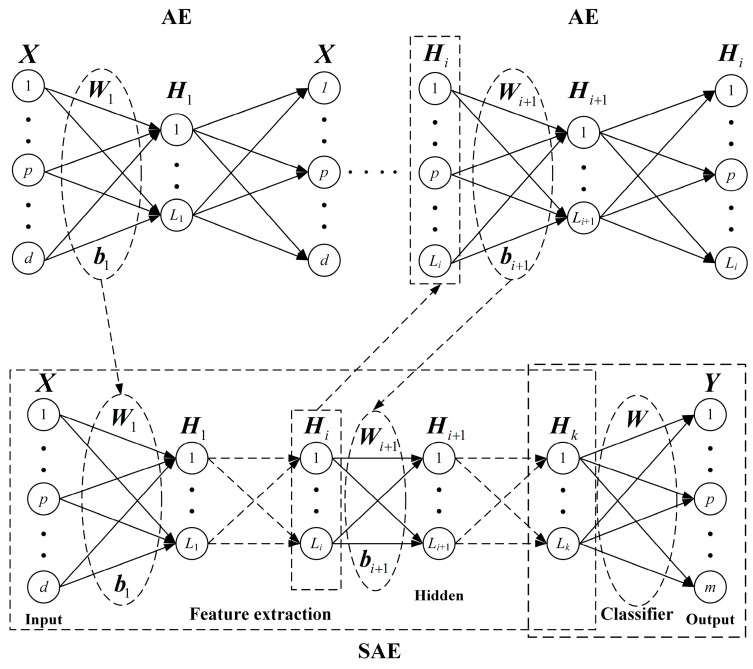
Network structure of the SAE.

The SAE is a supervised algorithm and consists of a stacked AE and a classifier. The AE extracts the hierarchical feature layer by layer, and the classifier maps the final abstract feature to the output. The SAE usually has a symmetric structure and includes encoding and decoding. However, the decoding process is often removed, and the final feature representation is used for classification and regression tasks. Suppose the training samples are $\left\{X,Y\right\}={\left\{\left(x_{i},y_{i}\right)\right\}}_{i=1}^{N}$, the network structure is $d,L_{1},L_{2},\ldots ,L_{k},m$ and the activation function is g(⋅). The network structure of the SAE is shown in Figure 2.

The SAE needs pre-training and fine-tuning to train the network. The pre-training stage determines the initial network parameters and extracts abstract features through the greedy layer-wise training of the AE. The fine-tuning stage computes the classification error and optimizes the network parameters.

In pre-training, the output $H_{i}$ of the ith hidden layer is the input of the AE, and the input weights $W_{i+1}$ and bias $b_{i+1}$ of the (i+1)th hidden layer can be obtained by the trained AE. $H_{k}$ is the final extracted feature and it is used as the input of the classifier to compute the output weights W and complete the classification mapping.

In fine-tuning, the SAE requires the calculation of the classification error of the training samples and backpropagates the error to optimize the network parameters using the gradient descent algorithm. When the cross-entropy error is used, the network loss function of the SAE is expressed as follows:

(4) $$J_{SAE}=-\frac{1}{N}\sum_{i=1}^{N}\sum_{c=1}^{m}p_{ic}log\left({\hat{y}}_{ic}\right)$$

where ${\hat{y}}_{ic}$ is the predicted probability that the ith sample belongs to class c, and $p_{ic}$ is the sign function. If the true label of the ith sample is c, $p_{ic}=1$, otherwise $p_{ic}=0$.

## 3. Semi-Supervised Stacked Autoencoder Based on the Pseudo Label

### 3.1. Pseudo Label

Traditional SAE can extract abstract features from labeled samples and complete the prediction or classification. However, the supervised learning of the SAE is only applicable to labeled samples, and unlabeled samples in the training data cannot be effectively utilized. The SAE is unable to use the feature and category information from unlabeled samples, and this limits the generalization performance of the SAE for semi-supervised tasks. Therefore, the PL-SSAE aims to propose a new semi-supervised SAE by introducing the pseudo-labeling method into the SAE. The PL-SSAE uses unlabeled samples for feature extraction and classification by generating the pseudo label and adding a regularization loss. The PL-SSAE makes full use of the feature and category information contained in unlabeled samples to improve the generalization performance of the SAE for semi-supervised problems. Compared with the SAE, the innovations of the PL-SSAE are the pre-training of the unlabeled samples, the generation of the pseudo label, and the construction of the pseudo-label regularization.

As a new approach to semi-supervised learning, the pseudo-labeling method aims to employ the network trained on labeled samples to predict unlabeled samples. Based on the clustering hypothesis, the most probable results are utilized as the pseudo labels for the unlabeled samples and the network is retrained with the pseudo-labeled samples. Thus, the application of the pseudo-labeling method requires three steps. The first step is to train the network with labeled samples. The second step is to predict the class of unlabeled samples and generate the pseudo label. The third step is to retrain the network with labeled and pseudo-labeled samples. The pseudo-labeling method represents both the labeling of the unlabeled samples and the semi-supervised training process of the network. Compared with other semi-supervised learning methods, the pseudo-labeling method can effectively exploit the category information contained in the unlabeled samples and improve the semi-supervised prediction and classification performance.

### 3.2. Network Structure

**Figure 3:**
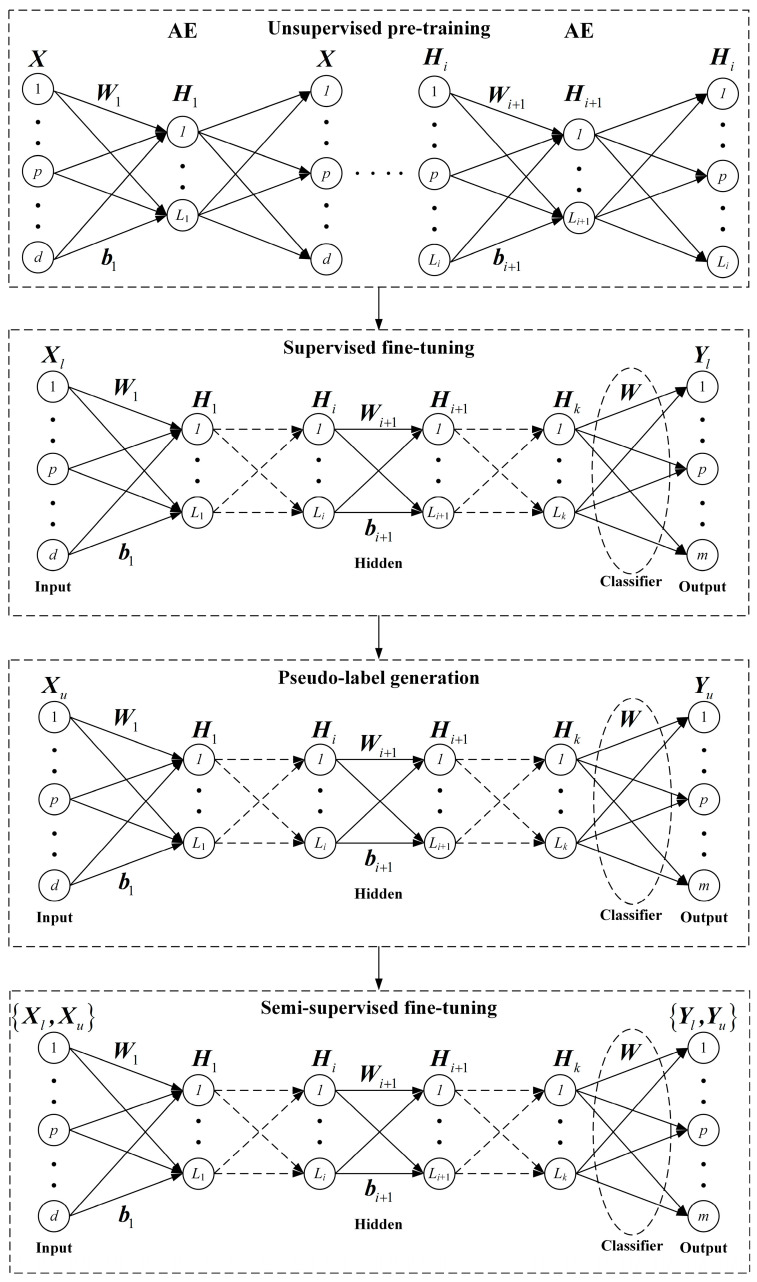
Network framework of the PL-SSAE.

According to the requirements of the pseudo-labeling method and the SAE, the PL-SSAE divides the first step of the pseudo-labeling method into unsupervised pre-training and supervised fine-tuning. Suppose that the labeled samples are $\left\{X_{l},Y_{l}\right\}$, the unlabeled samples are $X_{u}$, the network structure is $d,L_{1},L_{2},\ldots ,L_{k},m$ and the activation function is g(⋅). The network framework of the PL-SSAE is shown in Figure 3. The PL-SSAE consists of four stages: unsupervised pre-training, supervised fine-tuning, pseudo-label generation and semi-supervised fine-tuning.

In the unsupervised pre-training, similar to the SAE, the PL-SSAE trains the AE to assign network parameters layer by layer. However, unlike the SAE, the PL-SSAE requires both labeled and unlabeled samples in pre-training to fully exploit the feature information contained in the unlabeled samples. Meanwhile, using all samples at this stage avoids the repeated pre-training of pseudo-labeled samples and reduces the computational complexity. The relationship between the output of the ith hidden layer and the output of the (i+1)th hidden layer is expressed as follows:

(5) $$H_{i}=\left\{ \begin{array}{l} g\left(W_{1}X+b_{1}\right),i=1\\ g\left(W_{i}H_{i-1}+b_{i}\right),1<i\leq k \end{array}\right.$$

where $W_{i}$ and $b_{i}$ are the input weights and bias of the ith hidden layer, respectively, and $X=\left\{X_{l},X_{u}\right\}$ is the set of labeled and unlabeled samples. Through the greedy layer-wise pre-training, the PL-SSAE obtains the connection weights and biases of all hidden layers to achieve the assignment of network parameters.

In the supervised fine-tuning, the PL-SSAE calculates the classification loss of the labeled samples and optimizes the parameters of the pre-trained network. For the labeled samples $X_{l}$, the PL-SSAE obtains their predicted labels ${\hat{Y}}_{l}$ through feature extraction and classification. The classification loss between the predicted labels and the true labels is calculated by Formula (4), and the connection weights ${\left\{W_{i}\right\}}_{i=1}^{k}$ and bias ${\left\{b_{i}\right\}}_{i=1}^{k}$ of each hidden layer are adjusted by the stochastic gradient descent algorithm to determine the mapping function. The mapping function from the samples to the labels is formulated as follows:

(6) $$f:f\left(X\right)=Wg\left(W_{k}g\left(\ldots \left(W_{2}g\left(W_{1}X+b_{1}\right)+b_{2}\right)\ldots \right)+b_{k}\right)$$

In the pseudo-label generation, the PL-SSAE predicts the labels and determines the pseudo labels of the unlabeled samples with the supervised, trained network. For the unlabeled samples ${\left\{x_{i}\right\}}_{i=1}^{N}$, their prediction probabilities on different classes ${\left\{y_{i}^{j}\right\}}_{j=1}^{m}$ are calculated through forward propagation and label mapping. The label with the highest prediction probability is taken as the pseudo label of each unlabeled sample by the following formula:

(7) $$y_{i}=\underset{j,1\leq j\leq m}{\mathrm{argmax}\;}y_{i}^{j},i=1,2,\ldots ,N$$

In semi-supervised fine-tuning, the PL-SSAE inputs the labeled samples $\left\{X_{l},Y_{l}\right\}$ and pseudo-labeled samples $\left\{X_{u},Y_{u}\right\}$ into the network and computes the classification loss to optimize the network parameters. Since the pseudo labels are not necessarily the true labels of the unlabeled samples, the PL-SSAE introduces a regularization parameter to keep the loss balance between the labeled and pseudo-labeled samples. Using the cross-entropy function as a measure of the classification loss, the classification loss of the labeled samples $J_{l}$, the classification loss of the unlabeled samples $J_{u}$ and the total loss of the network $J_{PL-SSAE}$ are expressed as follows:

(8) $$J_{l}=-\frac{1}{N_{l}}\sum_{i=1}^{N_{l}}\sum_{c=1}^{m}p_{ic}^{l}log\left({\hat{y}}_{ic}^{l}\right)$$

(9) $$J_{u}=-\frac{1}{N_{u}}\sum_{j=1}^{N_{u}}\sum_{c=1}^{m}p_{jc}^{u}log\left({\hat{y}}_{jc}^{u}\right)$$

(10) $$J_{PL-SSAE}=J_{l}+\lambda J_{u}$$

where $N_{l}$ and $N_{u}$ are the number of the labeled and unlabeled samples, respectively, ${\hat{y}}_{ic}^{l}$ and ${\hat{y}}_{ic}^{u}$ are the prediction probabilities of the ith labeled sample and the jth unlabeled samples belonging to the class c. $p_{ic}^{l}$ and $p_{jc}^{u}$ are both the sign functions. If the true label of the ith labeled sample is c, $p_{ic}^{l}=1$, otherwise $p_{ic}^{l}=0$. If the pseudo label of the jth pseudo-labeled sample is c, $p_{jc}^{u}=1$, otherwise $p_{jc}^{u}=0$. The classification loss of the pseudo-labeled samples is the regularization term in the loss function to prevent over-fitting. By optimizing the network parameters, the network loss is gradually reduced, and the PL-SSAE classifies the labeled and pseudo-labeled samples more accurately.

### 3.3. Training Process

According to the network framework, the training process of the PL-SSAE consists of four stages: unsupervised pre-training, supervised fine-tuning, pseudo-label generation and semi-supervised fine-tuning. In the unsupervised pre-training stage, the network parameters are initialized by greedy layer-wise training on the labeled and unlabeled samples. In the supervised fine-tuning stage, the classification loss of the labeled samples is calculated, and the network parameters are optimized by the stochastic gradient descent algorithm. In the pseudo-label generation stage, the trained network predicts the class of the unlabeled samples and assigns pseudo labels to them. In the semi-supervised fine-tuning stage, the classification loss of the labeled and pseudo-labeled samples is computed to adjust the network parameters and complete the network training. Algorithm 1 presents the training details of the PL-SSAE.
**Algorithm 1:** Training process of the PL-SSAE.Input: The labeled samples $\left\{X_{l},Y_{l}\right\}$, the unlabeled samples $X_{u}$, the number of hidden nodes ${\left\{L_{i}\right\}}_{i=1}^{k}$, the regularization parameter λ, the number of mini-batch sizes s, the number of iteration t, learning rate α, and the activation function g(⋅)
Output: The mapping function $f:R^{d}\to R^{m}$.**The unsupervised pre-training**1:  **for** i=1 **to** k **do**2:    if i=1
3:     Let $X=\left\{X_{l},X_{u}\right\}$ be the input and output of the first AE4:    else5:     Let $H_{i-1}$ be the input and output of the ith AE6:    Randomly initialize the network parameters of the ith AE7:    **for** j=1 **to** t **do**8:     Obtain mini-batch samples ${\left\{x_{r}\right\}}_{r=1}^{s}$ from the input sample9:     Compute the hidden output $H_{i}$ of the AE by Equation (1)10:      Calculate the reconstructed samples ${\left\{{\hat{x}}_{r}\right\}}_{r=1}^{s}$ by Equation (2)11:      Compute the reconstruction loss of the AE by Equation (3)12:      Update the network parameters based on the stochastic gradient descent algorithm13:    **end for**14:    Assign the network parameters $\left\{W_{i},b_{i}\right\}$ of the ith AE to the ith hidden layer15:    Calculate the output $H_{i}$ of the ith hidden layer by Equation (5)16:  **end for****The supervised fine-tuning**17:  Input the labeled samples $\left\{X_{l},Y_{l}\right\}$ into the network18:  **for** j=1 **to** t **do**19:    Obtain mini-batch samples ${\left\{x_{r}\right\}}_{r=1}^{s}$ from the input samples20:    Predict the labels ${\left\{{\hat{y}}_{r}\right\}}_{r=1}^{s}$ of the mini-batch samples by Equation (6)21:    Calculate the classification loss by Equation (4)22:    Update the network parameters ${\left\{W_{i},b_{i}\right\}}_{i=1}^{k}$ and W based on the stochastic gradient descent algorithm23:  **end for****The pseudo-label generation**24:  Input the unlabeled samples $X_{u}$ into the network25:  Compute the class prediction of the unlabeled samples by Equation (6)26:  Generate the pseudo labels $Y_{u}$ of the unlabeled samples by Equation (7)**The semi-supervised fine-tuning**27:  Input the labeled samples $\left\{X_{l},Y_{l}\right\}$ and the pseudo-labeled samples $\left\{X_{u},Y_{u}\right\}$ into the network28:  **for** j=1 **to** t **do**29:    Obtain mini-batch samples ${\left\{x_{r}\right\}}_{r=1}^{s}$ from the input samples30:    Compute the class prediction of the input samples by Equation (6)31:    Calculate the total classification loss $J_{PL-SSAE}$ by Equations (8)–(10)32:    Update the network parameters ${\left\{W_{i},b_{i}\right\}}_{i=1}^{k}$ and W
33:  **end for**34:  **return** the mapping function $f:f\left(X\right)=Wg\left(W_{k}g\left(\ldots \left(W_{2}g\left(W_{1}X+b_{1}\right)+b_{2}\right)\ldots \right)+b_{k}\right)$


## 4. Experiments

To verify the semi-supervised classification performance of the proposed PL-SSAE, the following evaluations were designed and carried out:

Experiment 1: Influence of different hyperparameters. Observe the accuracy change in the PL-SSAE with a variable regularization parameter, variable percentage of labeled samples and variable number of hidden nodes, then analyze their influence on the classification performance of the PL-SSAE.

Experiment 2: Comparison of semi-supervised classification. Record the classification accuracy of the SAE, SSAE, Semi-SAE, Semi-SSAE and PL-SSAE with different percentages of labeled samples and compare the semi-supervised learning capability of different algorithms.

Experiment 3: Comparison of comprehensive performance. Observe the accuracy, precision, F1-measure, G-mean, training time and testing time of the SAE, SSAE, Semi-SAE, Semi-SSAE and PL-SSAE to compare their generalization performance and computational complexity.

### 4.1. Experimental Settings

#### 4.1.1. Data Description

Various benchmark datasets used in the evaluations are Rectangles, Convex, USPS [28], MNIST [29] and Fashion-MNIST [30]. The datasets are taken from the UCI Machine Learning Repository [31] and have been normalized to [0,1]. Details of the benchmark datasets are shown in Table 1.

#### 4.1.2. Implementation Details

All evaluations were carried out in Pytorch 1.9, running on a desktop with a 3.6 GHz Intel 12,700 K CPU, Nvidia RTX 3090 graphics, 32 GB RAM and a 2 TB hard disk. To avoid the uncertainty and ensure a fair comparison, all reported results are the averages of 20 repeated experiments, and the same network structure is utilized for different algorithms. The network structure of each algorithm used in Experiments 2 and 3 is shown in Table 2.

The experimental details of Experiment 1 are as follows: The dataset is MNIST, the batch size is 100, the number of iterations is 100, the learning rate is 0.01 and the activation function is a ReLU function. Suppose parameter p represents the percentage of labeled samples in the training data. When changing the regularization parameter and the percentage of labeled samples, the network structure is 784-300-200-100-10, the range of regularization parameter is λ∈{0,0.1,0.2,…,1} and the range of label percentage is p∈{5,10,15,…,50}. When changing the number of hidden nodes, the network structure is $784-L_{1}-L_{2}-10$, the range of $L_{1}$ is $L_{1}\in \left\{100,200,\ldots ,900,1000\right\}$, the range of $L_{2}$ is $L_{2}\in \left\{100,200,\ldots ,900,1000\right\}$, the regularization parameter is λ=0.5 and the label percentage is p=20.

The experimental details for Experiment 2 are as follows: The datasets are Convex, USPS, MNIST and Fashion-MNSIT. The batch size is 100, the number of iterations is 100, the learning rate is 0.01, the activation function is a ReLU function and the range of the label percentage is p∈{5,10,15,…,50}. The sparsity parameter of the SSAE and Semi-SSAE is ρ=0.05, and the regularization parameter of the PL-SSAE is λ=0.5.

The experimental details for Experiment 3 are as follows: The batch size is 100, the number of iterations is 100, the learning rate is 0.01, the activation function is the ReLU function, and the range of label percentage is p∈{5,10,15,20}. The sparsity parameter of the SSAE and Semi-SSAE is ρ=0.05 and the regularization parameter of the PL-SSAE is λ=0.5. For multiclass classification tasks, the precision, F1-measure and G-mean are the averages of different classes.

### 4.2. Influence of Different Hyperparameters

**Figure 4:**
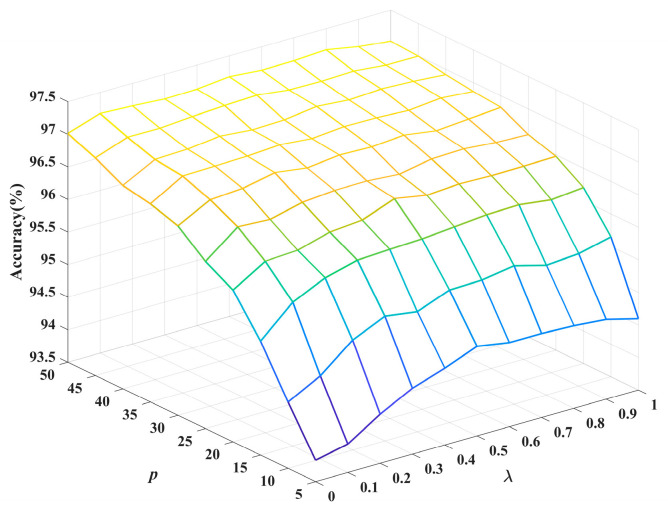
The influence of the regularization parameter and label percentage on the generalization performance.

**Figure 5:**
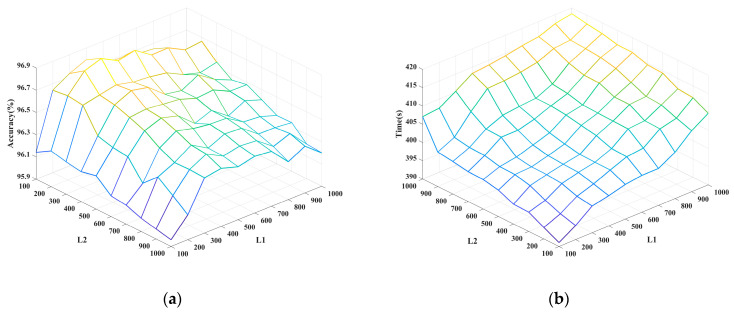
The influence of the hidden nodes on (**a**) accuracy and (**b**) training time.

As predetermined parameters of the network, the hyperparameters affect the semi-supervised learning and classification performance of the PL-SSAE. The regularization parameter, the percentage of labeled samples and the number of hidden nodes are important hyperparameters for the PL-SSAE. The regularization parameter controls the balance between the empirical loss and the regularization loss. The percentage of labeled samples determines the number of labeled and pseudo-labeled samples. The number of hidden nodes controls the structural complexity and fitting ability of the network. To analyze the specific influence of different hyperparameters, a variable regularization parameter, a variable percentage of labeled samples and a variable number of hidden nodes are utilized to observe the accuracy change in the PL-SSAE. The generalization performance of the PL-SSAE with different regularization parameters and label percentages is shown in Figure 4. The generalization performance and training time of the PL-SSAE with different numbers of hidden nodes are shown in Figure 5.

As is shown in Figure 4, the semi-supervised classification performance of the PL-SSAE varies with the regularization parameter and the percentage of labeled samples. When the label percentage p is fixed, the classification accuracy of the PL-SSAE increases and then decreases as the regularization parameter λ increases. When the regularization parameter λ is fixed, the classification accuracy increases as the label percentage p increases. This is because the regularization parameter λ controls the importance of the pseudo-label loss in the loss function. A proper regularization parameter λ allows the PL-SSAE to exploit the feature and category information contained in unlabeled samples to improve its semi-supervised learning. However, an excessively large λ will cause the PL-SSAE to ignore the labeled samples, and the difference between the pseudo labels and the true labels will lead to an under-fitting. Therefore, it is important to choose appropriate regularization parameters for different samples. However, the trial-and-error method used for regularization parameter selection in the PL-SSAE is time-consuming and inefficient. Meanwhile, the labeled samples are the prior knowledge for the network. With the increase in label percentage p, the number of labeled samples in the training data increases and more category information improves the generalization performance of the network.

As is shown in Figure 5, the classification accuracy and training time of the PL-SSAE vary with the number of hidden nodes. As the number of hidden nodes increases, the generalization performance of the PL-SSAE increases and then decreases. The reason is that the hidden nodes control the function approximation ability of the network. As the number of hidden nodes increases, the generated pseudo labels are closer to the true labels and more category information contained in pseudo-labeled samples improves the semi-supervised learning of the PL-SSAE. However, too many hidden nodes will lead to the over-fitting of the network, and the difference between the training and testing samples will cause the classification accuracy to decrease. In addition, the training time of the PL-SSAE increases with the increase in hidden nodes. This is because the number of hidden nodes is positively correlated with the computational complexity of the network. When the computational power is fixed, the increase in the computational complexity leads to an increase in the training time.

### 4.3. Comparison of Semi-Supervised Classification

**Figure 6:**
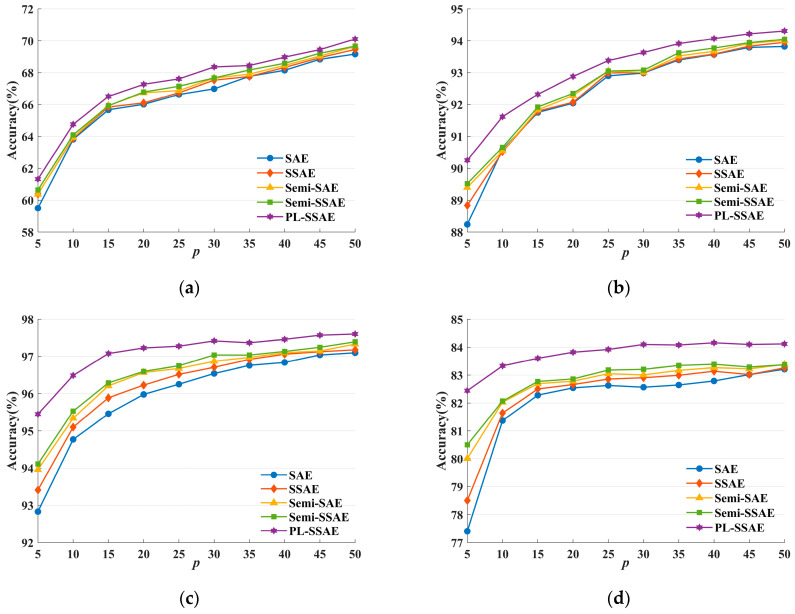
Comparison of the semi-supervised classification on (**a**) Convex, (**b**) USPS, (**c**) MNIST and (**d**) Fashion-MNIST datasets.

The semi-supervised classification performance is a direct reflection of the ability to learn from unlabeled training samples. To evaluate the semi-supervised classification performance of different algorithms, it is necessary to adopt different percentages of labeled samples, then record the accuracy change on the testing samples and plot the accuracy curves. The experiment in this section focuses on comparing the PL-SSAE with the SAE, SSAE, Semi-SAE and Semi-SSAE. The variation in classification accuracy of each algorithm on datasets with different label percentages is shown in Figure 6.

As shown in Figure 6, the semi-supervised classification performance of the PL-SSAE outperforms that of the SAE, SSAE and Semi-SAE and Semi-SSAE on different datasets. As the label percentage increases, the number of labeled training samples increases. Thus, more label information is exploited to learn the function mapping, and the generalization performance of each algorithm gradually increases. The classification accuracy of the PL-SSAE is higher than other algorithms at different label percentages. The reason is that the PL-SSAE is an effective semi-supervised algorithm. Compared with the supervised SAE and SSAE, the PL-SSAE uses the feature information and category information of the unlabeled samples to make the learned mapping function closer to the real mapping. Compared with the Semi-SAE and Semi-SSAE, the PL-SSAE not only utilizes the unlabeled samples for feature extraction but also exploits the pseudo-label information for classification mapping. The advantage of the PL-SSAE in the semi-supervised classification becomes more apparent when the percentage of labeled samples is small. However, when there are sufficient labeled samples, PL-SSAE tends to have no performance advantage and the inconsistency between pseudo labels and true labels will reduce the generalization performance of the PL-SSAE.

### 4.4. Comparison of Comprehensive Performance

To test the comprehensive performance of the PL-SSAE, all benchmark datasets mentioned above are used to compare the PL-SSAE with the SAE, SSAE, Semi-SAE and Semi-SSAE. Different metrics, such as accuracy, precision, F1-measure and G-mean, of each algorithm with different label percentages are recorded to evaluate the semi-supervised performance. The training and testing times of each algorithm are recorded to compare the computational complexity. The classification accuracy, precision, F1-measure, G-mean, training time and testing time of each algorithm are shown in Table 3, Table 4, Table 5, Table 6, Table 7 and Table 8, respectively (the numbers in bold indicate the best results). Since the experimental results are the averages of repeated experiments, the standard deviation of the results is listed after the average to reflect the performance stability of the algorithm.

As shown in Table 3, Table 4, Table 5 and Table 6 the comprehensive performance of the PL-SSAE is better than that of the SAE, SSAE, Semi-SAE and Semi-SSAE. For each dataset, the PL-SSAE has higher classification accuracy, precision, F1-measure and G-mean than other algorithms with different label percentages. The reason is that the SAE and SSAE do not use unlabeled samples in the training process, and the Semi-SAE and Semi-SSAE only use unlabeled samples in the feature extraction process. The PL-SSAE introduces the pseudo label and makes appropriate use of the labeled samples to generate the pseudo labels of the unlabeled samples. The category information contained in the pseudo-labeled samples guides the feature extraction and class mapping of the network, and this improves the semi-supervised learning and classification performance of the PL-SSAE. Moreover, the PL-SSAE integrates the pseudo-label regularization into the loss function. The balance between the classification loss of the labeled and pseudo-labeled samples avoids over-fitting and improves the generalization performance.

As shown in Table 7 and Table 8, the training time of PL-SSAE is slightly higher than that of the SAE, SSAE, Semi-SAE and Semi-SSAE, while the testing time of each algorithm is the same. The PL-SSAE requires additional fine-tuning of the pseudo-labeled samples. As a result, the computational complexity and training time of the PL-SSAE is twice that of the other algorithms. However, given the improvement in generalization performance, the increase in training time for the PL-SSAE is worthwhile. In the comparison of testing speed, the testing time is related to the sample size and network structure. Therefore, different algorithms with the same testing samples and network structure have the same testing speed.

## 5. Conclusions

To overcome the limitations of traditional SAE for unlabeled samples, this study integrates the pseudo label into the SAE and proposes a new semi-supervised SAE called PL-SSAE. The PL-SSAE assigns the pseudo labels to the unlabeled samples by the network trained on the labeled samples and adds a pseudo-label regularization term to the loss function. Different from the SAE, the PL-SSAE exploits the feature and category information contained in the unlabeled samples to guide the feature extraction and classification of the network. Various evaluations on different datasets show that the PL-SSAE outperforms the SAE, SSAE, Semi-SAE and Semi-SSAE.

However, the different hyperparameters of the PL-SSAE in this study are determined by the time-consuming trial-and-error method. Thus, it is important to combine the PL-SSAE with the particle swarm optimization algorithm [32] or the ant colony algorithm [33] to achieve automatic optimization of the hyperparameters. In addition, the PL-SSAE only determines the pseudo labels by taking the maximum value of the prediction probabilities. This method tends to introduce noise. Therefore, a more effective method needs to be investigated to further generate more reasonable pseudo labels.

## Figures and Tables

**Table 1:** The datasets used in the experiments.

Datasets	Attributes	Classes	Training Data	Testing Data
Rectangles	784	2	1200	50,000
Convex	784	2	8000	50,000
USPS	256	10	7291	2007
MNIST	784	10	60,000	10,000
Fashion-MNIST	784	10	60,000	10,000

**Table 2:** The network structure of SAE, SSAE, Semi-SAE, Semi-SSAE and PL-SSAE.

Datasets	Network Structure				
	SAE	SSAE	Semi-SAE	Semi-SSAE	PL-SSAE
Rectangles	784-200-100-2				
Convex	784-200-100-2				
USPS	256-200-100-10				
MNIST	784-400-200-100-10				
Fashion-MNIST	784-200-100-50-10				

**Table 3:** The accuracy comparison of SAE, SSAE, Semi-SAE, Semi-SSAE and PL-SSAE.

Datasets		Accuracy (%)				
		SAE	SSAE	Semi-SAE	Semi-SSAE	PL-SSAE
Rectangles	*p* = 5	62.06 ± 0.68	62.72 ± 0.72	62.85 ± 0.83	62.86 ± 0.52	**64.65 ± 0.40**
	*p* = 10	64.41 ± 0.55	64.95 ± 0.72	65.24 ± 0.53	65.33 ± 0.38	**66.24 ± 0.27**
	*p* = 15	71.61 ± 1.25	71.73 ± 1.17	72.12 ± 1.40	72.20 ± 1.12	**73.17 ± 1.05**
	*p* = 20	74.28 ± 1.32	74.61 ± 1.05	74.95 ± 0.85	75.23 ± 1.03	**76.01 ± 1.13**
Convex	*p* = 5	59.50 ± 0.55	60.39 ± 0.60	60.38 ± 0.63	60.64 ± 0.60	**61.32 ± 0.46**
	*p* = 10	63.82 ± 0.46	63.89 ± 0.67	63.93 ± 0.89	64.10 ± 0.51	**64.76 ± 0.64**
	*p* = 15	65.67 ± 0.86	65.84 ± 0.81	65.74 ± 0.61	65.92 ± 0.49	**66.51 ± 0.66**
	*p* = 20	66.01 ± 0.61	66.10 ± 0.54	66.54 ± 0.29	66.79 ± 0.42	**67.27 ± 0.35**
USPS	*p* = 5	88.24 ± 0.24	88.84 ± 0.35	89.40 ± 0.22	89.52 ± 0.10	**90.25 ± 0.10**
	*p* = 10	90.59 ± 0.39	90.52 ± 0.32	90.54 ± 0.24	90.66 ± 0.23	**91.62 ± 0.33**
	*p* = 15	91.75 ± 0.22	91.79 ± 0.20	91.83 ± 0.22	91.92 ± 0.06	**92.32 ± 0.18**
	*p* = 20	92.04 ± 0.19	92.07 ± 0.13	92.28 ± 0.19	92.35 ± 0.11	**92.88 ± 0.11**
MNIST	*p* = 5	92.83 ± 0.20	93.41 ± 0.07	93.97 ± 0.17	94.11 ± 0.09	**95.45 ± 0.12**
	*p* = 10	94.77 ± 0.11	95.10 ± 0.12	95.34 ± 0.11	95.53 ± 0.10	**96.49 ± 0.11**
	*p* = 15	95.46 ± 0.07	95.89 ± 0.10	96.21 ± 0.15	96.29 ± 0.06	**97.08 ± 0.08**
	*p* = 20	95.98 ± 0.13	96.23 ± 0.10	96.57 ± 0.11	96.60 ± 0.11	**97.23 ± 0.04**
Fashion-MNIST	*p* = 5	77.41 ± 1.47	78.50 ± 1.26	80.01 ± 1.57	80.50 ± 1.33	**82.44 ± 1.52**
	*p* = 10	81.37 ± 1.12	81.64 ± 0.97	82.03 ± 1.15	82.07 ± 0.91	**83.33 ± 1.07**
	*p* = 15	82.28 ± 0.54	82.50 ± 0.57	82.69 ± 0.67	82.77 ± 0.61	**83.60 ± 0.60**
	*p* = 20	82.54 ± 0.71	82.66 ± 0.68	82.78 ± 0.38	82.86 ± 0.41	**83.82 ± 0.64**

Results in bold are better than other algorithms.

**Table 4:** The precision comparison of SAE, SSAE, Semi-SAE, Semi-SSAE and PL-SSAE.

Datasets		Precision (%)				
		SAE	SSAE	Semi-SAE	Semi-SSAE	PL-SSAE
Rectangles	*p* = 5	62.95 ± 1.36	63.30 ± 1.12	63.70 ± 1.09	63.89 ± 1.30	**65.20 ± 0.64**
	*p* = 10	65.11 ± 1.49	65.10 ± 1.32	65.98 ± 0.95	65.67 ± 0.98	**67.14 ± 0.78**
	*p* = 15	71.61 ± 1.01	71.05 ± 0.99	72.46 ± 1.29	72.31 ± 1.13	**73.71 ± 1.36**
	*p* = 20	73.34 ± 1.80	73.92 ± 1.73	74.04 ± 1.15	74.10 ± 1.03	**75.42 ± 1.03**
Convex	*p* = 5	58.38 ± 1.05	58.96 ± 1.05	59.13 ± 1.00	59.19 ± 0.97	**60.44 ± 0.82**
	*p* = 10	61.55 ± 0.77	61.58 ± 1.16	61.99 ± 1.07	62.10 ± 1.07	**62.74 ± 1.38**
	*p* = 15	64.93 ± 0.95	64.94 ± 1.09	64.96 ± 0.82	65.12 ± 0.74	**65.61 ± 0.53**
	*p* = 20	65.35 ± 0.98	65.95 ± 1.19	65.41 ± 1.21	66.03 ± 0.93	**66.76 ± 0.98**
USPS	*p* = 5	88.22 ± 0.26	88.51 ± 0.28	88.75 ± 0.24	88.86 ± 0.16	**89.55 ± 0.06**
	*p* = 10	89.91 ± 0.43	89.87 ± 0.36	89.97 ± 0.28	90.07 ± 0.23	**91.23 ± 0.39**
	*p* = 15	91.06 ± 0.28	91.12 ± 0.36	91.19 ± 0.21	91.35 ± 0.11	**91.76 ± 0.26**
	*p* = 20	91.45 ± 0.23	91.53 ± 0.25	91.77 ± 0.21	91.85 ± 0.12	**92.26 ± 0.18**
MNIST	*p* = 5	92.92 ± 0.21	93.66 ± 0.07	93.93 ± 0.18	94.06 ± 0.10	**95.38 ± 0.13**
	*p* = 10	94.75 ± 0.12	95.06 ± 0.11	95.31 ± 0.10	95.51 ± 0.08	**96.47 ± 0.12**
	*p* = 15	95.46 ± 0.06	95.87 ± 0.10	96.19 ± 0.16	96.27 ± 0.06	**97.05 ± 0.08**
	*p* = 20	95.95 ± 0.14	96.20 ± 0.09	96.55 ± 0.11	96.57 ± 0.11	**97.23 ± 0.05**
Fashion-MNIST	*p* = 5	77.35 ± 1.56	78.08 ± 1.08	79.86 ± 1.36	80.35 ± 1.20	**82.20 ± 1.04**
	*p* = 10	81.43 ± 1.30	82.00 ± 1.00	82.09 ± 1.44	82.37 ± 1.07	**83.48 ± 1.10**
	*p* = 15	82.38 ± 0.65	82.55 ± 0.58	82.79 ± 0.56	82.94 ± 0.77	**83.68 ± 0.79**
	*p* = 20	82.52 ± 0.70	82.74 ± 0.79	82.87 ± 0.58	82.91 ± 0.60	**83.95 ± 0.75**

Results in bold are better than other algorithms.

**Table 5:** The F1-measure comparison of SAE, SSAE, Semi-SAE, Semi-SSAE and PL-SSAE.

Datasets		F1-Measure (%)				
		SAE	SSAE	Semi-SAE	Semi-SSAE	PL-SSAE
Rectangles	*p* = 5	60.48 ± 1.08	60.50 ± 0.86	60.73 ± 0.67	60.90 ± 0.72	**62.85 ± 0.65**
	*p* = 10	63.59 ± 1.07	64.37 ± 1.04	64.59 ± 1.35	64.93 ± 1.28	**66.19 ± 1.49**
	*p* = 15	71.16 ± 0.86	72.26 ± 1.07	72.16 ± 1.15	72.54 ± 0.98	**73.62 ± 1.02**
	*p* = 20	74.79 ± 1.29	74.97 ± 1.31	75.40 ± 0.98	75.79 ± 1.38	**76.56 ± 0.83**
Convex	*p* = 5	62.17 ± 1.09	62.98 ± 1.48	62.99 ± 1.10	62.98 ± 1.21	**63.70 ± 0.98**
	*p* = 10	65.83 ± 1.34	66.20 ± 1.22	66.06 ± 1.53	66.84 ± 1.21	**67.50 ± 1.04**
	*p* = 15	66.69 ± 1.02	68.09 ± 1.34	67.84 ± 0.81	68.14 ± 0.96	**68.89 ± 0.92**
	*p* = 20	67.85 ± 0.80	68.50 ± 0.89	68.94 ± 1.13	69.06 ± 0.76	**69.74 ± 0.82**
USPS	*p* = 5	87.22 ± 0.38	87.84 ± 0.78	88.37 ± 0.27	88.58 ± 0.13	**89.28 ± 0.12**
	*p* = 10	89.77 ± 0.43	89.71 ± 0.36	89.75 ± 0.27	89.88 ± 0.24	**90.97 ± 0.37**
	*p* = 15	91.00 ± 0.26	91.02 ± 0.25	91.10 ± 0.24	91.16 ± 0.08	**91.56 ± 0.21**
	*p* = 20	91.30 ± 0.23	91.29 ± 0.20	91.57 ± 0.21	91.64 ± 0.16	**92.20 ± 0.15**
MNIST	*p* = 5	92.87 ± 0.20	93.63 ± 0.07	93.89 ± 0.18	94.04 ± 0.10	**95.40 ± 0.12**
	*p* = 10	94.71 ± 0.12	95.14 ± 0.11	95.29 ± 0.11	95.48 ± 0.10	**96.42 ± 0.13**
	*p* = 15	95.40 ± 0.06	95.86 ± 0.10	96.17 ± 0.16	96.26 ± 0.06	**97.05 ± 0.08**
	*p* = 20	95.93 ± 0.14	96.29 ± 0.10	96.54 ± 0.11	96.56 ± 0.11	**97.22 ± 0.03**
Fashion-MNIST	*p* = 5	76.37 ± 1.69	77.45 ± 1.13	79.17 ± 1.27	79.87 ± 1.44	**81.44 ± 1.70**
	*p* = 10	81.02 ± 1.08	81.56 ± 1.04	81.64 ± 1.32	81.80 ± 0.63	**82.22 ± 0.96**
	*p* = 15	81.96 ± 0.55	82.19 ± 0.65	82.34 ± 0.87	82.59 ± 0.91	**83.01 ± 0.77**
	*p* = 20	82.17 ± 0.88	82.29 ± 0.46	82.69 ± 0.49	82.77 ± 0.36	**83.76 ± 0.53**

Results in bold are better than other algorithms.

**Table 6:** The G-mean comparison of SAE, SSAE, Semi-SAE, Semi-SSAE and PL-SSAE.

Datasets		G-Mean (%)				
		SAE	SSAE	Semi-SAE	Semi-SSAE	PL-SSAE
Rectangles	*p* = 5	95.97 ± 0.12	96.42 ± 0.05	96.57 ± 0.10	96.66 ± 0.05	**97.43 ± 0.07**
	*p* = 10	97.03 ± 0.07	97.29 ± 0.07	97.36 ± 0.06	97.47 ± 0.06	**98.01 ± 0.08**
	*p* = 15	97.43 ± 0.04	97.65 ± 0.06	97.86 ± 0.09	97.91 ± 0.04	**98.36 ± 0.04**
	*p* = 20	97.73 ± 0.07	97.94 ± 0.05	98.07 ± 0.07	98.08 ± 0.06	**98.45 ± 0.02**
Convex	*p* = 5	86.87 ± 0.70	87.52 ± 0.86	88.45 ± 0.94	88.74 ± 0.90	**89.94 ± 0.94**
	*p* = 10	89.27 ± 0.67	89.43 ± 0.58	89.66 ± 0.69	89.68 ± 0.47	**90.28 ± 0.91**
	*p* = 15	89.81 ± 0.32	89.96 ± 0.37	90.05 ± 0.40	90.11 ± 0.36	**90.62 ± 0.48**
	*p* = 20	89.96 ± 0.43	90.36 ± 0.40	90.64 ± 0.23	90.76 ± 0.25	**91.15 ± 0.37**
USPS	*p* = 5	61.93 ± 0.57	62.30 ± 0.96	62.25 ± 1.10	62.38 ± 0.88	**64.36 ± 0.23**
	*p* = 10	64.29 ± 0.48	64.87 ± 0.72	64.94 ± 0.59	65.27 ± 0.41	**65.80 ± 0.38**
	*p* = 15	71.40 ± 1.07	71.53 ± 0.88	71.74 ± 0.87	71.89 ± 0.99	**72.77 ± 1.21**
	*p* = 20	74.22 ± 1.33	74.54 ± 1.03	74.90 ± 1.09	75.13 ± 1.00	**76.24 ± 0.97**
MNIST	*p* = 5	58.64 ± 1.10	59.81 ± 1.01	59.73 ± 0.77	59.94 ± 0.75	**60.47 ± 0.49**
	*p* = 10	62.89 ± 0.61	62.92 ± 1.17	63.09 ± 1.04	63.29 ± 0.97	**64.10 ± 1.04**
	*p* = 15	65.05 ± 1.08	65.27 ± 0.86	65.23 ± 0.68	65.34 ± 1.10	**66.39 ± 0.87**
	*p* = 20	65.69 ± 0.78	65.69 ± 0.83	65.98 ± 0.55	66.24 ± 0.48	**66.81 ± 0.23**
Fashion-MNIST	*p* = 5	92.64 ± 0.17	93.03 ± 0.18	93.37 ± 0.17	93.58 ± 0.05	**93.89 ± 0.12**
	*p* = 10	94.27 ± 0.23	94.23 ± 0.16	94.24 ± 0.14	94.31 ± 0.16	**94.89 ± 0.21**
	*p* = 15	94.99 ± 0.16	94.98 ± 0.11	95.03 ± 0.16	95.04 ± 0.05	**95.32 ± 0.12**
	*p* = 20	95.11 ± 0.14	95.21 ± 0.12	95.24 ± 0.12	95.29 ± 0.10	**95.72 ± 0.08**

Results in bold are better than other algorithms.

**Table 7:** The training time of SAE, SSAE, Semi-SAE, Semi-SSAE and PL-SSAE.

Datasets		Training Time (s)				
		SAE	SSAE	Semi-SAE	Semi-SSAE	PL-SSAE
Rectangles	*p* = 5	4.307 ± 0.501	4.551 ± 1.131	5.530 ± 0.001	6.199 ± 0.876	11.833 ± 0.672
	*p* = 10	4.697 ± 0.536	4.887 ± 0.066	5.695 ± 0.459	6.608 ± 0.911	12.321 ± 0.812
	*p* = 15	4.989 ± 0.596	4.700 ± 0.020	5.965 ± 0.694	6.693 ± 0.679	12.432 ± 0.813
	*p* = 20	5.136 ± 0.496	5.185 ± 0.499	6.288 ± 0.640	6.758 ± 0.378	13.065 ± 1.012
Convex	*p* = 5	5.790 ± 0.091	5.779 ± 0.476	17.188 ± 0.835	19.968 ± 0.747	37.163 ± 2.359
	*p* = 10	7.635 ± 0.481	7.785 ± 0.440	19.104 ± 0.805	21.013 ± 0.719	39.100 ± 2.732
	*p* = 15	9.367 ± 0.454	9.802 ± 0.729	20.317 ± 0.880	22.618 ± 0.983	41.352 ± 2.340
	*p* = 20	11.757 ± 0.476	12.191 ± 0.975	22.115 ± 0.836	24.155 ± 0.910	43.395 ± 2.210
USPS	*p* = 5	1.793 ± 0.163	1.828 ± 0.157	9.151 ± 0.563	11.218 ± 0.350	20.096 ± 0.991
	*p* = 10	3.156 ± 0.278	3.713 ± 0.709	10.136 ± 0.788	11.989 ± 1.230	20.803 ± 1.714
	*p* = 15	4.689 ± 0.621	4.800 ± 0.930	11.104 ± 1.000	12.959 ± 0.794	21.658 ± 1.142
	*p* = 20	5.999 ± 0.522	6.321 ± 1.148	11.951 ± 0.987	14.367 ± 0.796	22.060 ± 1.392
MNIST	*p* = 5	18.957 ± 0.896	17.142 ± 1.472	149.063 ± 2.258	172.881 ± 1.375	367.162 ± 2.683
	*p* = 10	36.686 ± 0.738	35.380 ± 1.557	160.620 ± 2.823	183.834 ± 1.573	384.812 ± 3.075
	*p* = 15	51.161 ± 1.554	54.447 ± 1.151	173.278 ± 2.472	194.655 ± 2.311	398.623 ± 3.822
	*p* = 20	79.166 ± 1.570	82.322 ± 1.539	186.880 ± 2.461	209.533 ± 2.135	434.780 ± 2.810
Fashion-MNIST	*p* = 5	16.120 ± 0.635	17.557 ± 0.902	149.418 ± 1.191	172.441 ± 0.942	368.544 ± 2.431
	*p* = 10	33.148 ± 1.178	35.654 ± 1.837	160.116 ± 1.692	184.956 ± 1.280	380.945 ± 2.933
	*p* = 15	49.904 ± 1.304	53.817 ± 1.138	171.385 ± 1.382	197.478 ± 1.815	397.639 ± 3.021
	*p* = 20	77.729 ± 1.622	81.660 ± 1.800	189.076 ± 0.940	212.044 ± 2.509	437.919 ± 2.892

**Table 8:** The testing time of SAE, SSAE, Semi-SAE, Semi-SSAE and PL-SSAE.

Datasets		Testing Time (s)				
		SAE	SSAE	Semi-SAE	Semi-SSAE	PL-SSAE
Rectangles	*p* = 5	0.050 ± 0.007	0.046 ± 0.001	0.046 ± 0.001	0.046 ± 0.001	0.047 ± 0.001
	*p* = 10	0.051 ± 0.007	0.047 ± 0.002	0.047 ± 0.002	0.047 ± 0.002	0.047 ± 0.001
	*p* = 15	0.042 ± 0.004	0.046 ± 0.001	0.047 ± 0.001	0.047 ± 0.001	0.046 ± 0.001
	*p* = 20	0.047 ± 0.006	0.046 ± 0.001	0.047 ± 0.002	0.047 ± 0.001	0.046 ± 0.002
Convex	*p* = 5	0.039 ± 0.007	0.042 ± 0.002	0.040 ± 0.001	0.040 ± 0.001	0.040 ± 0.001
	*p* = 10	0.038 ± 0.006	0.040 ± 0.001	0.039 ± 0.001	0.039 ± 0.001	0.041 ± 0.001
	*p* = 15	0.047 ± 0.010	0.040 ± 0.001	0.040 ± 0.002	0.040 ± 0.001	0.039 ± 0.001
	*p* = 20	0.042 ± 0.008	0.042 ± 0.004	0.039 ± 0.001	0.040 ± 0.002	0.040 ± 0.001
USPS	*p* = 5	0.008 ± 0.001	0.005 ± 0.001	0.005 ± 0.001	0.005 ± 0.001	0.004 ± 0.001
	*p* = 10	0.008 ± 0.002	0.005 ± 0.002	0.005 ± 0.001	0.005 ± 0.001	0.005 ± 0.002
	*p* = 15	0.006 ± 0.001	0.005 ± 0.001	0.005 ± 0.002	0.004 ± 0.001	0.004 ± 0.001
	*p* = 20	0.007 ± 0.001	0.005 ± 0.001	0.005 ± 0.001	0.005 ± 0.002	0.005 ± 0.001
MNIST	*p* = 5	0.017 ± 0.002	0.013 ± 0.003	0.012 ± 0.004	0.014 ± 0.001	0.012 ± 0.002
	*p* = 10	0.014 ± 0.002	0.014 ± 0.001	0.015 ± 0.007	0.012 ± 0.004	0.012 ± 0.001
	*p* = 15	0.016 ± 0.002	0.013 ± 0.003	0.012 ± 0.005	0.013 ± 0.003	0.013 ± 0.002
	*p* = 20	0.016 ± 0.001	0.014 ± 0.001	0.014 ± 0.001	0.013 ± 0.003	0.015 ± 0.003
Fashion-MNIST	*p* = 5	0.014 ± 0.001	0.014 ± 0.002	0.013 ± 0.003	0.014 ± 0.001	0.012 ± 0.001
	*p* = 10	0.013 ± 0.003	0.014 ± 0.001	0.012 ± 0.004	0.014 ± 0.001	0.014 ± 0.002
	*p* = 15	0.014 ± 0.001	0.014 ± 0.001	0.014 ± 0.004	0.014 ± 0.001	0.014 ± 0.001
	*p* = 20	0.013 ± 0.004	0.013 ± 0.003	0.012 ± 0.006	0.014 ± 0.002	0.014 ± 0.003

## Data Availability

The data used to support the findings of this study are available from the corresponding author upon request.
